# Efficacy of silymarin in patients with non-alcoholic fatty liver disease — the Siliver trial: a study protocol for a randomized controlled clinical trial

**DOI:** 10.1186/s13063-023-07210-6

**Published:** 2023-03-10

**Authors:** Camila Ribeiro de Avelar, Beatriz Vieira Coelho Nunes, Betina da Silva Sassaki, Mariana dos Santos Vasconcelos, Lucivalda Pereira Magalhães de Oliveira, André Castro Lyra, Allain Amador Bueno, Rosângela Passos de Jesus

**Affiliations:** 1 Department of Nutrition Sciences, Federal University of Bahia, Bahia, 32 Araújo Pinho Street, Canela, Salvador, Bahia 40.110-150 Brazil; 2 Gastrohepatology Service, Professor Edgard Santos University Hospital, Federal University of Bahia, Salvador, Bahia Brazil; 3 College of Health, Life and Environmental Sciences, University of Worcester, Worcester, WR2 6AJ UK

**Keywords:** Clinical trial, Silymarin, Non-alcoholic fatty liver disease, randomized controlled trial

## Abstract

**Background:**

Non-alcoholic fatty liver disease (NAFLD) is one of the most prevalent liver diseases globally. Pharmacological treatments for NAFLD are still limited. Silymarin, a compound extracted from *Silybum marianum*, is an herbal supplement traditionally used in folk medicine for liver disorders. It has been proposed that silymarin may possess hepatoprotective and anti-inflammatory properties. The present trial aims to assess the efficacy of silymarin supplementation in the adjuvant treatment of NAFLD in adult patients.

**Method:**

This is a randomized double-blind placebo-controlled clinical trial recruiting adult NAFLD patients in therapy on an outpatient basis. Participants are randomized to an intervention (I) or control (C) group. Both groups receive identical capsules and are followed for 12 weeks. I receives 700mg of silymarin + 8mg vitamin E + 50mg phosphatidylcholine daily, while C receives 700mg maltodextrin + 8mg vitamin E + 50mg phosphatidylcholine daily. Patients undergo a computerized tomography (CT) scan and blood tests at the beginning and end of the study. Monthly face-to-face consultations and weekly telephone contact are carried out for all participants. The primary outcome assessed will be change in NAFLD stage, if any, assessed by the difference in attenuation coefficient between liver and spleen, obtained by upper abdomen CT.

**Discussion:**

The results of this study may provide a valuable opinion on whether silymarin can be used as adjuvant therapy for the management or treatment of NAFLD. The data presented on the efficacy and safety of silymarin may provide more foundation for further trials and for a possible use in clinical practice.

**Trial registration:**

This study has been approved by the Research Ethics Committee of the Professor Edgard Santos University Hospital Complex, Salvador BA, Brazil, under protocol 2.635.954. The study is carried out according to guidelines and regulatory standards for research involving humans, as set out in Brazilian legislation. Trial registration - ClinicalTrials.gov : NCT03749070. November 21, 2018

## Strengths and limitations of this study

1. Placebo-controlled randomized double-blind clinical trial employing silymarin extract with greater bioavailability.
2. The effectiveness of silymarin supplementation will be assessed by CT without contrast as reference standard for the detection and assessment of liver steatosis [1].
3. Participants are recruited from a Nutrition and Hepatology Clinic of a single tertiary referral hospital.

## Introduction

### Background and rationale {6a}

NAFLD is one of the most prevalent liver diseases worldwide. With a continuously increased incidence and level of complications, NAFLD has become a major public health concern worldwide [2–4], with approximately 20% to 30% of the general adult population affected. Men appear to show a higher prevalence for NAFLD than women in all age groups [5]. Based on risk factors, NAFLD is manifested in approximately 50% of overweight individuals and in approximately 80% to 90% of obese individuals. Individuals diagnosed with metabolic syndrome (MS) are approximately twice as likely to develop NAFLD [6]. The main risk factors associated with NAFLD overlap with those of metabolic syndrome, including central obesity, type 2 diabetes (T2D), dyslipidemia, and insulin resistance (IR). NAFLD has been associated with a pro-inflammatory background and is considered a hepatic manifestation of obesity and MS [7, 8].

In its first stage, NAFLD patients show lipid inclusion in the liver parenchyma without evident signs of inflammation or hepatocellular necrosis. At this stage, NAFLD management is focused on improving IR, body fat reduction, as well as MS and T2D prevention and management. Body weight reduction combined with amelioration of metabolic disarrangements can prevent the progression of steatosis to non-alcoholic steatohepatitis (NASH), cirrhosis, and cellular hepatocarcinoma [9, 10].

Despite our understanding of the epidemiological and pathophysiological aspects of NAFLD, the main and by far most successful treatment option available is a positive lifestyle change. The use of pharmacological agents is still limited and requires stronger evidence regarding safety and efficacy [11]. As successful long-term adherence to positive lifestyle changes is not always achieved in full, researchers have investigated supporting pharmacotherapeutic strategies, such as herbal medicines, to ameliorate NAFLD. A few studies have suggested that silymarin supplementation may induce beneficial effects for NAFLD patients, including amelioration of biochemical markers associated with inflammation and NAFLD progression [12–15].

Silymarin is a flavonoid extracted from *Silybum marianum*, one of the most used medicinal herbs by individuals with liver diseases [16]. *Silybum marianum* has shown good tolerance and safety, with limited adverse effects reported in liver disease patients. The hepatoprotective, anti-inflammatory, antioxidant, and anti-fibrotic effects of silymarin have been studied in patients with cirrhosis associated with viral hepatitis, exposure to environmental toxins, alcoholic steatosis and NASH [17–20].

A few studies [13, 16, 18] have pointed out to a beneficial effect of silymarin therapy upon the evolution of NAFLD, but significant variability and methodological differences across available studies prevent the establishment of robust conclusions. A systematic review with meta-analysis [16] including six clinical trials showed that silymarin reduced serum levels of alanine aminotransferase (ALT) and aspartate aminotransferase (AST) in NAFLD patients, but the studies appraised in that meta-analysis had a high degree of heterogeneity and low methodological quality. The scarcity of clinical trials employing silymarin as adjuvant therapy for liver disease and NAFLD, with a particular attention to methodological design, planning, and execution stages, has encouraged us to propose and execute the Siliver trial. Our initial hypothesis is that silymarin supplementation will improve the metabolic status and reduce liver fat content in NAFLD patients. Our hypothesis will be tested by following the protocol detailed below.

### Objectives {7}

The present study aims to investigate the efficacy of silymarin supplementation as an adjunctive treatment for adult patients suffering with NAFLD. The primary outcome assessed will be change in NAFLD stage, if any, assessed by the difference in attenuation coefficient between liver and spleen, obtained by upper abdomen CT scan without contrast. The attenuation coefficient between the liver and spleen is a reference standard for the assessment of liver steatosis.

As secondary objectives, we will assess body mass index (BMI) and waist circumference (WC); glucose metabolism biomarkers including blood glucose, insulin, glycated hemoglobin (HbA1C), and Homeostasis Model Assessment-Insulin Resistance Index (HOMA-IR); blood ferritin levels; and biomarkers of liver damage, including transaminases, gamma-glutamyl transferase (γGT), and alkaline phosphatase (AP).

### Trial design {8}

**Fig. 1:**
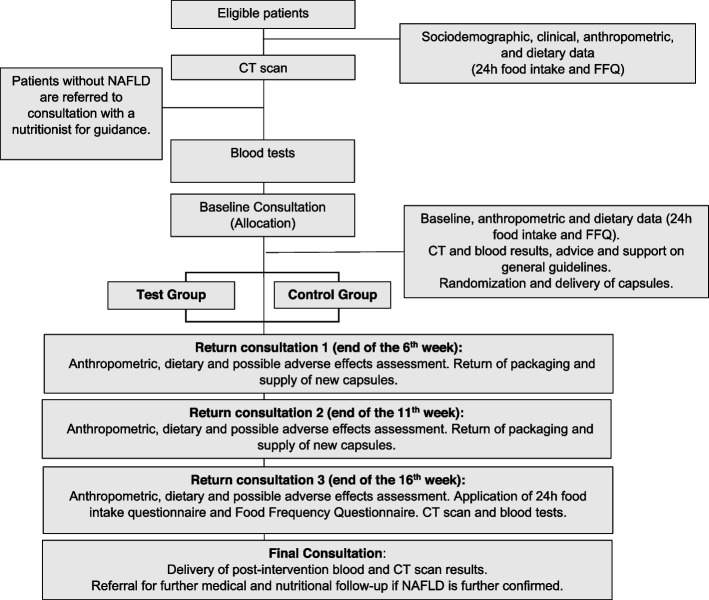
Study flowchart

As the trial hypothesis is to investigate whether silymarin supplementation is better than placebo for NAFLD amelioration, a framework of superiority was adopted for this double-blind randomized placebo-controlled clinical trial. Patients will be supplemented for 12 weeks (Fig. 1). The planning of this trial follows the guidelines of the Consolidated Standards of Reporting Trials (CONSORT) and Standard Protocol Items: Recommendations for Interventional Trials (SPIRIT) guidelines.

## Method: participants, interventions, and outcomes

### Study settings {9}

All participants had a confirmed NAFLD diagnosis prior to participation in this trial. NAFLD patients were referred to the trial by local health centers located in the neighborhoods of the city of Salvador BA Brazil, in partnership with our outpatient recruitment unit at the Nutrition and Hepatology Outpatient Clinic of the Professor Edgard Santos University Hospital Complex (NHOC), located in the city of Salvador BA, Brazil, where the trial took place.

### Eligibility criteria {10}

#### Inclusion criteria

Eligibility criteria for the Siliver trial include consenting adult patients aged between 20 and 60 years of both sexes. All patients had a medically confirmed diagnosis of NAFLD prior to participation in this trial.

#### Non-inclusion criteria

Patients who meet any of the following criteria have not been included lactating or pregnant women, and women during their menacme, except for those who underwent definitive contraception such as hysterectomy or tubal ligation; patients with a previously established diagnosis of chronic disease including congestive heart failure, decompensated or severe lung disease, neoplasms, kidney disease, carriers of the human immunodeficiency virus (HIV), and advanced chronic liver diseases (Child-Pugh classification B and C) caused by any causal agent unrelated to NAFLD; recreational drug users; patients with an average intake of more than 20 g of alcohol per day and or a history of alcoholic disease in abstention for less than 6 months; use of prescribed medication including steroids, oestrogens, amiodarone, warfarin, anticonvulsants, antipsychotics, or chemotherapy drugs in the past 6 months; infections, surgery, trauma, or hospitalization in the past 30 days; chronic degenerative diseases of non-hepatic origin; and unavailability of previously obtained imaging tests with a diagnosis of NAFLD in its screening phase.

#### Exclusion criteria

The following patients will be excluded patients who at the recruitment stage do not show confirmation of NAFLD by CT scan; patients who missed any phase of the trial; and patients who during the trial were diagnosed with a condition listed in the non-inclusion criteria above.

### Who will provide informed consent? {26a}

Patients will provide informed consent themselves. All eligible participants have received information about the study and had the opportunity to have their questions answered to their satisfaction. The blinded evaluator has obtained written informed consent (IC) from all participants prior to assessment.

### Additional consent provisions for collection and use of participant data and biological specimens {26b}

The blood samples obtained, once analyzed for the specific purpose of this trial, are discarded following the Teaching Hospital Standard Operating Procedures (SOPs) on disposal of biological waste. Data collected from participants withdrawn from the study or lost on follow-up will be excluded and deleted, as the aim of the trial has not been completed for those participants. All participants have given their consent to the research team to share relevant data with researchers taking part in the research, as well as regulatory authorities. This set of information was explained to participants and made available in the consent form. All participants have agreed to the above.

## Interventions

### Explanation for the choice of comparators {6b}

Participants are randomized to groups intervention (I) or control (C). They receive identical capsules and follow the trial instructions for 12 weeks. All participants are instructed to take two capsules per day soon after a meal. Considering the pathophysiology of NAFLD and pharmacotherapies currently available for its treatment or management, there are no specific pharmacological agents currently approved for NAFLD specifically that could be compared to the test item. Therefore, the research team adopted a methodological design using a placebo.

### Intervention description {11a}

I participants receive 700 mg silymarin + 8 mg vitamin E + 50 mg phosphatidylcholine, daily. C participants receive 700 mg maltodextrin + 8 mg vitamin E + 50 mg phosphatidylcholine, daily.

Each I capsule contains 350 mg silymarin + 4 mg vitamin E + 25 mg phosphatidylcholine. Each C capsule contains 350 mg maltodextrin + 4 mg vitamin E + 25 mg phosphatidylcholine. All participants are instructed to take two capsules per day soon after a meal.

We have carefully defined the capsule composition for both groups to achieve a planned daily dosage. We employed phosphatidylcholine and vitamin E in the composition to increase silymarin bioavailability, which is based on a protocol described previously and evaluated in a systematic review with meta-analysis published by our group [15].

Microcrystalline cellulose, corn starch, and colloidal silicon dioxide were used as standard excipients in all capsules of both I and C. The capsule composition did not include dyes, preservatives or additives in I or C, guaranteeing the standardization of their appearance. The capsule weight, size, shape, and coating were identical between I and C, and have been designed to ensure the same ease of swallowing, to minimize risks of gastric discomfort for the participants, and to ensure similar disintegration time and propensity for swelling. The capsules are visually unidentifiable between I and C. All I and C capsules were manufactured and kindly donated by Singular Pharma (Salvador, Brazil). The expiry date is three months after production.

### Criteria for discontinuing or modifying allocated interventions {11b}

Participants are free to withdraw from the trial at any time and for any reason without penalty. Participants are withdrawn from the study if they start a different treatment elsewhere.

### Strategies to improve adherence to interventions {11c}

Participants are monitored weekly by telephone calls to collect information on adherence. They are given the opportunity to ask the research team any question and receive reminders of upcoming appointments. In addition, participants are instructed to bring along the latest flasks with the capsules received at each appointment, favoring greater adherence and commitment to the treatment.

### Relevant concomitant care permitted or prohibited during the trial {11d}

During the intervention and follow-up, participants are advised not to use any medication, supplement, tea or herbal supplement without prior medical and nutritional advice, and without prior notice to the research team. If they do, they are asked to inform the research team immediately, informing what was ingested and the date and dosage taken.

### Provisions for post-trial care {30}

All study participants have the right to medical care and follow-up, and if they experience a worsening of their condition. In the final consultation, participants who no longer show fatty infiltration receive guidance on how to prevent the recurrence of NAFLD. Participants who show evidence of NAFLD, regardless of the degree of steatosis, are referred to the outpatient follow-up clinic for the continuation of their care provision with a hepatologist consultant and a nutritionist (Fig. 1).

### Outcomes {12}

#### Primary outcome

The primary outcome will be the assessment of NAFLD resolution, or change in its grade, as assessed by the difference in the attenuation coefficient between liver and spleen, obtained by CT of the upper abdomen, at the end (after) compared to beginning (before) the trial. In summary, the primary outcome is to investigate whether silymarin supplementation can reduce liver fat content in NAFLD patients, measured by a CT scan.

#### Secondary outcomes

The secondary outcomes investigated in this trial include:

- Differences in ALT, AST, γGT, and AP levels after *versus* before;
- Difference in ferritin levels after *versus* before;
- Difference in fasting glucose, insulin, HbA1C, and HOMA-IR levels after *versus* before; and
- Difference in BMI and WC after *versus* before.

### Participant timeline {13}

Table 1 shows the stages of the study and which assessments will be performed throughout the study period.

**Table 1:** Schedule of enrolment, interventions, and assessments

Schedule of activities									
	Enrolment	Allocation	Post-allocation					Close-out	
Time Point	Week 1	Week 2	Week 3–5	Week 6	Week 7–10	Week 11	Week 12–15	Week 16	Week 17
**Enrolment**									
Screening	•								
Eligibility screen	•								
Informed consent	•								
Computed tomography (CT) and collection of laboratory tests	•								
Baseline consultation (allocation)		•							
**Interventions**									
Intervention start		•							
Telephone follow-up			•		•		•		
Return consultation 1				•					
Return consultation 2						•			
Return consultation 3								•	
**Assessments**									
Computed tomography (CT) and laboratory tests								•	
Final consultation									•

### Sample Size {14}

The sample size was determined through an accuracy analysis of the primary outcome. A significance level of 0.05 and a power of 80% were adopted, which resulted in a minimum sample size of approximately 132 participants.

### Recruitment {15}

Patient recruitment started in February 2019 and ended in May 2022. All participants have been diagnosed with NAFLD prior to participation in this trial. All participants were recruited directly from the NHOC, or they have been referred to the NHOC by local health centers located in the neighborhoods of the city of Salvador. The NHOC provides specialist care for patients with liver diseases, including NAFLD. However, only patients diagnosed with NAFLD have been approached to discuss their participation in the trial.

All patients received in May 2022 or onwards a phone call with an invitation for a face-to-face consultation, in which their individual results will be discussed with a clinician researcher. Any current or new need for medical care will be discussed at that consultation, and a new referral will be made if necessary.

The burden of this randomized controlled trial was not assessed by the patients themselves or their families. Prior to signing the consent forms, all patients were reassured that their participation in this trial was entirely voluntary. All patients were also reassured that they could withdraw at any time without giving a reason and reassured that withdrawing would not result in any penalty to them and would not interfere with any current or future medical or healthcare provision to them.

## Intervention assignment: allocation

### Sequence generation {16a}

Patients who agreed to take part in the study and met all the study criteria were assigned to I or C groups by computer-generated randomization in blocks with the aid of a spreadsheet to ensure a random but uniform variation in sizes of each I and C blocks. Randomization and record keeping of all participants were carried out by a statistician researcher external to the research group. The statistician is not involved in patient clinical assessment.

### Concealment mechanism {16}

The allocation sequence was concealed from the researchers assessing participants and their clinical outcomes. The allocation sheet hardcopies are kept in envelopes in a locked drawer of a secure filing cabinet located at the NHOC administrative office.

### Implementation {16c}

All patients who give consent for participation and who fulfill the inclusion criteria are randomly assigned to I or C. The Principal Investigator (PI) will open the envelopes containing the allocation sequence only after the last participant included completes the trial.

## Assignment of interventions: blinding

### Who will be blinded {17a}

All clinical assessments were conducted by evaluators blinded to treatment allocation. Participants were blinded to the study hypothesis and their allocation group, but were advised of the overall aspects involved in the treatment of both groups. I and C capsules were dispensed by a registered pharmacist at the clinical research pharmacy of the Teaching Hospital where the trial took place. The pharmacist was responsible for receiving and storing all batches of I and C capsules manufactured by Singular Pharma, as well as identifying the correct capsule to be dispensed according to the specific numbering of each participant.

### Procedure for unblinding if needed {17b}

The trial design is open-label with outcome assessors being blinded. Only the pharmacist in charge of dispensing the trial capsules and the statistician have access to group allocation; neither, however, are involved in clinical assessment or data collection.

### Data collection and management {18a}

The research team involved in this trial underwent training prior to commencement of the study regarding the protocol to be followed, to avoid biases and errors in data collection. The clinical dietitians (CRA, BVCN, BSS, MSV) in charge of seeing patients at the NHOC performed a triage of eligible patients, and the more experienced dietitian running the clinic (CRA) was responsible for double-checking that all the inclusion and non-inclusion criteria had been fully met for each patient. CRA was also responsible for explaining the study in further detail and taking verbal and written consent. The clinical trial is structured as presented in Table 1 and detailed below.

#### Screening

Patients who meet the eligibility criteria are referred to the screening consultation to receive information about the clinical trial and are given the opportunity to ask questions to the research team. Consenting participants are invited to sign the free and informed consent form. All participants are informed that they can withdraw at any time without giving reasons and that they will not be penalized for withdrawing. After signing the consent form, all participants answer questions on a standardized form filled in by the researcher. Information on sociodemographics, clinical history and clinical presentation, lifestyle data, diet intake, and feeding patterns are collected. The first anthropometric assessment is completed at that point.

At the end of the screening consultation, participants receive general nutritional guidelines and a study identification card with the contact details of the researchers. Participants are encouraged to make contact should they have any late questions about the trial. Following the first appointment, participants are referred to an upper abdomen CT scan before the intervention begins.

##### CT scans

Participants undergo a scheduled upper abdomen CT scan at the Radiology Unit of the Professor Edgard Santos University Hospital Complex. NAFLD is confirmed by a radiologist consultant. Patients who do not present NAFLD at the CT scan, even if they have historical imaging tests diagnosing the disease at an earlier period, are excluded from this trial and referred to a consultation with a registered nutritionist to receive specific guidelines on how to prevent NAFLD recurrence,

##### Blood tests

After the CT scan, participants are taken a 12-h fasting venous blood sample. They are given a breakfast meal after the blood sample collection and are given the details of their next consultation.

#### Allocation consultation

At this appointment, participants receive their CT scan and blood test results and are submitted to a second anthropometric assessment. At this consultation, a new form with nutritional and dietary data is completed by the researcher, and patients are given additional nutritional guidelines to complement the advice offered in the screening appointment. Subsequently, participants are sent to the clinical research pharmacy of the Hospital Complex for capsule collection according to their randomization.

#### Monthly follow-up visits (return visits 1, 2, and 3)

All participants were followed up through face-to-face individual consultations in 4-week intervals during the 12-week supplementation period. The Return Visits were scheduled as detailed in Table 1. At these appointments, patients were asked about adherence to the study. They were asked to bring along to each consultation the flasks they receive containing the capsules from the previous 4-week period, so that the research team can more accurately estimate adherence to the protocol.

At each monthly follow-up visit participants had a nutritional and dietary assessment undertaken by a registered nutritionist member of the research team. The researcher completed a form with dietary intake and dietary habits over the last 4-week period and evaluated adherence to nutritional guidelines, ingestion of capsules as prescribed, tolerance, and the occurrence of any adverse effects.

#### Telephone follow-up

except for the weeks when participants had their scheduled Return Visits, all participants were contacted weekly by telephone to monitor adherence to the protocol, clarify any questions, and report any occurrence or adverse reactions.

At Return Visit 3, which took place at the end of the 12^th^ week of intervention, patients were referred to a second CT scan and blood tests following the same protocols as the baseline tests. A final consultation was scheduled for the test results to be delivered (Table 1).

In the final consultation, participants who no longer showed fatty infiltration received guidance on how to prevent the recurrence of NAFLD. Participants who showed evidence of NAFLD, regardless of the degree of steatosis, were referred to the outpatient follow-up clinic for continuation of their care provision with a hepatologist consultant and a registered nutritionist (Fig. 1).

### Plans to promote participant retention and complete follow-up {18b}

Participants received information about the study design and the importance of completing the final follow-up. The research team contacted the participants via telephone calls, and in case of not reaching, a voice message or text message 24 h before the scheduled appointments and blood tests, aiming to minimize non-attendance.

### Data management {19}

The data were collected in paper form and stored in binders, which are kept in a locked drawer of a secure filing cabinet located at the NHOC administrative office. Only the PI has access to the documents.

After data collection, all forms will be checked by two members for data quality and missing information. The data will be entered manually into an electronic spreadsheet and subsequently checked by two researchers, one at a time.

The database and electronic analyses will be stored on a secure computer server with personal login access authorized by the PI. After completion of the study, all data and study documents will be archived and stored by the PI. The data is not public and remains in the possession of the PI. Individual unidentifiable data can be made available upon reasonable request.

### Confidentiality {27}

The data will be treated anonymously and confidentially and at no time will the personal details of the participants be disclosed at any stage of the study.

### Plans for collection, laboratory evaluation, and storage of biological specimens for genetic or molecular analysis in this trial/future use {33}

Blood tests were performed following standardized laboratory protocols for medical diagnosis in humans. Blood samples were not stored for genetic or molecular analysis in the current study or any other future use. Blood samples were discarded following standardized hospital protocol. Blood test results were collected from the clinical analysis laboratory of the Institute of Pharmacy of the Federal University of Bahia via a secure hospital intranet.

## Statistical methods

### Statistical methods for primary and secondary outcomes {20a}

Statistical analyses will firstly be performed using descriptive analysis to characterize the distribution of the events studied. Categorical variables will be investigated using simple absolute frequencies. Continuous variables will be investigated by measures of central tendency and dispersion. Parametric or non-parametric tests will be used considering the distribution nature of the variables studied. A significance level of 5% will be adopted for all statistical tests. Tests to verify variable behavior and comparisons of proportions analyses, such as the application of the chi-square test or Fisher’s exact test, will be discussed later with a qualified statistician. Tests for comparison of means between groups, analysis of correlations between continuous variables and logistic regression analysis, will also be discussed with the statistician. Data will be tabulated and analyzed using the R Project for Statistical Computing software (R-3.2.4 for Windows).

### Interim analyses {21b}

This is a low-risk intervention, as silymarin at the dosage adopted in this trial has been considered safe and well-tolerated. All patients were carefully followed up in person and by telephone, aiming to identify any adverse event. Only patients who successfully complete the trial will have their data included in the final data analyses.

### Methods for additional analyses (e.g., subgroup analyses) {20b}

No additional analysis will be performed.

### Methods in analysis to handle protocol non-adherence and any statistical methods to handle missing data {20c}

Only patients who successfully completed the trial will have their data included in the final trial data analyses, and there will be no imputations for missing data. The data will be assessed by intention-to-treat, in which all participants who completed the trial are included in the statistical analyses and analyzed according to the group they were originally assigned, regardless of which group they were assigned to. Participants who drop out of the study due to illness, moving to another city, or inability to attend appointments or perform tests will be considered as protocol deviations and will be excluded from the data analyses.

### Plans to give access to the full protocol, participant-level data, and statistical code {31c}

The full protocol, participant-level data, and statistical code generated in this trial will be available from the corresponding author in electronic format on reasonable request. Identifiable information such as full name, address, and date of birth will not be shared for confidentiality purposes.

## Oversight and monitoring

### Composition of the coordinating center and trial steering committee {5d}

The PI as a blind evaluator and a coordinator assistant will coordinate all phases of the study, the randomization, and record-keeping of I and C participants. Patient and Public Involvement Groups (PPIG) were not involved in the design, recruitment, or execution of this trial, nor will they be involved in the reporting and dissemination of this research trial, with the exception of dissemination through their own social media channels if they wish.

### Composition of the data monitoring committee, its role and reporting structure {21a}

There will be no data monitoring committee since only the primary evaluator, the research team, and the coordinator assistant will have access to the clinical trial data. Additionally, this trial is a low-risk intervention, and participants are advised to report any unexpected or adverse effects to the research team.

### Adverse event reporting and harms {22}

The capsules provided to patients in both groups were produced and kindly donated by Singular Pharma (Salvador BA, Brazil). Singular Pharma have contractually undertaken not to interfere in any stage of the trial and have allowed the dissemination of any trial results, even if such results do not confirm the hypothesis that silymarin may be a beneficial adjuvant compound for the treatment or management of NAFLD. The donation document was submitted to, and approved by, the Ethics Committee.

Silymarin is commonly prescribed by clinicians and nutritionists and its use is considered safe [15]. According to data available from studies included in a previously published meta-analysis [15], there have been no reports of serious adverse events categorized as frequent or uncommon. However, a few studies have reported episodes of nausea, vomiting, and abdominal discomfort as rare adverse events [16, 17, 20]. Interestingly, a few clinical trials and meta-analyses that have investigated the effects of supplements containing silymarin did not record adverse events or complications associated with supplementation, suggesting good tolerability and safety to participants [16, 17, 20].

In the current trial, participants were advised to immediately stop taking the capsules and contact the research team as soon as possible in the event of any unexpected or adverse effect, or any discomfort supposedly associated with the capsule intake. All participants were followed up and monitored every 4 weeks at the NHOC, where the data collection and weekly telephone follow-up calls took place. All possible adverse events or complications observed were recorded, evaluated, and reported in the study.

### Frequency and plans for auditing trial conduct {23}

Fortnightly meetings were held with the group of researchers involved in the study to discuss development and question clarification. An independent researcher external to the research team will verify the data collected during the study. If any documents are missing or information is inconsistent, the Ethics Committee will be notified. Lastly, if there is any change in the study, the ethics committee, the journal, and *ClinicalTrials* will be notified immediately.

### Plans for communicating important protocol amendments to relevant parties (e.g., trial participants, ethical committees) {25}

This study has been approved by the Research Ethics Committee of the Professor Edgard Santos University Hospital Complex under application protocol 2.635.954. The research must be undertaken as set out in the approved documents for the approval to be valid. The Research Ethics Committee will be contacted should the research team intend to make any amendments to the approved research.

Patients eligible to join the trial were invited to sign the consent form after receiving all the information regarding the trial, including potential risks, and after having their questions answered in full. The consent form was developed in compliance with guidelines and regulatory standards for research involving human beings. During all stages of this trial the standards set out in the Brazilian Resolutions 196 and 466/2012, approved by the National Health Council, were followed. The Good Clinical Practices of the Document of the Americas of 2008 were also followed.

All participant information is kept confidential. At the end of the trial, all participants were advised to maintain their clinical and nutritional follow-up appointments at the NHOC of the University Hospital Complex according to their health needs.

### Dissemination plans {31a}

The results of this randomized controlled clinical trial are expected to be disseminated through presentations at conferences and publications in peer-reviewed journals.

## Discussion

This clinical trial aims to assess the efficacy of silymarin in adult patients diagnosed with NAFLD. The prevalence and incidence of NAFLD are expanding at an accelerated pace and currently pose a burden to public health systems. There are currently limited pharmacotherapeutical options for NAFLD [3, 4].

Silymarin has been hypothesized as a useful adjuvant therapeutic resource in clinical practice for NAFLD therapies, as beneficial hepatoprotective properties attributed to silymarin have been reported [21]. A few clinical studies have observed improvement in liver function and injury biomarkers after silymarin supplementation in various liver diseases [14, 22–25]. Liver biomarker levels are a true representation of the progression of liver disease and their levels are strongly associated with greater morbidity. Amelioration of liver biomarkers attributed to silymarin therapy is of great benefit for sufferers and a promising tool worth of further investigation .

Several studies investigating the effects of silymarin in patients with liver diseases unfortunately feature some methodological biases. A meta-analysis of clinical trials published by our research team [15] found a high degree of heterogeneity and low methodological quality in the qualitative assessment of the clinical trials included in the meta-analysis. Lastly, only very few published studies recruiting reasonable sample sizes have evaluated the efficacy of silymarin supplemented as a single test item in adult patients with NAFLD; we have addressed such a problem in our study by supplementing silymarin only.

The lack of robust evidence, as well as inconsistencies identified in existing publications, reinforce the need for additional clinical trials with stronger methodological designs to further elucidate the roles of silymarin, if any, in the treatment and management of NAFLD. The results of the present clinical trial may contribute to the dissemination of reliable outcomes that may or may not support the recommendation of silymarin adjuvant therapy for NAFLD patients.

## Trial status

Patient recruitment started in February 2019 and ended in May 2022. A significant delay in study progression and data collection was attributed to the SARS-CoV-2 pandemic and subsequent lockdowns.

## Data Availability

Any data required to support the protocol can be supplied on request.

## Abbreviations

NAFLD: Non-alcoholic fatty liver disease
T: Test
C: Control
MS: Metabolic syndrome
T2D: Type 2 diabetes
IR: Insulin resistance
NASH: Non-alcoholic steatohepatitis
ALT: Alanine aminotransferase
AST: Aspartate aminotransferase
γGT: Gamma-glutamyl transferase
AP: Alkaline phosphatase
BMI: Body mass index
WC: Waist circumference
CONSORT: Consolidated Standards of Reporting Trials
SPIRIT: Standard Protocol Items- Recommendations for Interventional Trails
HIV: Human immunodeficiency vírus
IC: Informed consent

## References

[1] Cerit M, Şendur HN, Cindil E, Erbaş G, Yalçın M, Cerit E (2020). Quantification of liver fat content with ultrasonographic attenuation measurement function: correlation with unenhanced multidimensional computerized tomography. Clin Imaging.

[2] Dan AA, Kallman JB, Wheeler A, Younoszai Z, Collantes R, Bondini S (2007). Health-related quality of life in patients with non-alcoholic fatty liver disease. Aliment Pharmacol Ther.

[3] Beaton MD, Chakrabarti S, Levstik M, Speechley M, Marotta P, Adams P (2013). Phase II clinical trial of phlebotomy for non-alcoholic fatty liver disease. Aliment Pharmacol Ther.

[4] Nseir W, Hellou E, Assy N (2014). Role of diet and lifestyle changes in nonalcoholic fatty liver disease. World J Gastroenterol.

[5] Fresneda S, Abbate M, Busquets-Cortés C, López-González A, Fuster-Parra P, Bennasar-Veny M (2022). Sex and age differences in the association of fatty liver index-defined non-alcoholic fatty liver disease with cardiometabolic risk factors: a cross-sectional study. Biol Sex Differ.

[6] Veena J, Muragundla A, Sidgiddi S, Subramaniam S (2014). Non-alcoholic fatty liver disease: need for a balanced nutritional source. Br J Nutr.

[7] Younossi ZM, Stepanova M, Afendy M, Fang Y, Younossi Y, Mir H (2011). Changes in the prevalence of the most common causes of chronic liver diseases in the United States from 1988 to 2008. Clin Gastroenterol Hepatol.

[8] Bellentani S (2017). The epidemiology of non-alcoholic fatty liver disease. Liver Int.

[9] Fan JG, Jia JD, Li YM, Wang BY, Lu LG, Shi JP (2011). Guidelines for the diagnosis and management of nonalcoholic fatty liver disease: update 2010. J Dig Dis.

[10] Chitturi S, Wong VW, Chan WK, Wong GL, Wong SK, Sollano J (2018). The Asia–Pacific working party on non-alcoholic fatty liver disease guidelines 2017—part 2: management and special groups. J Gastroenterol Hepatol.

[11] Fan JG, Wei L, Zhuang H (2019). National workshop on fatty liver and alcoholic liver disease, Chinese society of hepatology, Chinese medical association; fatty liver disease expert committee, Chinese medical doctor association. Guidelines of prevention and treatment of nonalcoholic fatty liver disease (2018, China). J Dig Dis.

[12] Hashemi SJ, Hajiani E, Sardabi EH (2009). A placebo-controlled trial of silymarin in patients with nonalcoholic fatty liver disease. Hepat Mon.

[13] Masoodi M, Rezadoost AM, Panahian M, Vojdanian M (2013). Effects of silymarin on reducing liver aminotransferases in patients with nonalcoholic fatty liver diseases. Govaresh.

[14] Solhi H, Ghahremani R, Kazemifar AM, Hoseini YZ (2014). Silymarin in treatment of non-alcoholic steatohepatitis: a randomized clinical trial. Caspian J Intern Med.

[15] Fathalah WF, Abdel Aziz MA, Abou El Soud NH, El Raziky MES (2017). High dose of silymarin in patients with decompensated liver disease: A randomized controlled trial. J Interf Cytokine Res.

[16] De Avelar CR, Pereira EM, de Farias Costa PR, de Jesus RP, de Oliveira LPM (2017). Effect of silymarin on biochemical indicators in patients with liver disease: Systematic review with meta-analysis. World J Gastroenterol.

[17] Rainone F (2005). Milk thistle. Am Fam Physician.

[18] Saller R, Brignoli R, Melzer J, Meier R (2008). An updated systematic review with meta-analysis for the clinical evidence of silymarin. Forsch Komplementmed.

[19] Milosevic N, Milanovic M, Abenavoli L, Natasa M (2014). Phytotherapy and NAFLD – from goals and challenges to clinical practice. Rev Recent Clin Trials.

[20] Yang Z, Zhuang L, Lu Y, Xu Q, Chen X. Effects and tolerance of silymarin (Milk Thistle) in chronic hepatitis C virus infection patients: A meta-analysis of randomized controlled trials. Biomed Res Int. 2014:941085. 10.1155/2014/941085. 10.1155/2014/941085 PMC4163440 25247194

[21] Schrieber SJ, Hawke RL, Wen Z, Smith PC, Reddy KR, Wahed AS (2011). Differences in the disposition of silymarin between patients with nonalcoholic fatty liver disease and chronic hepatitis C. Drug Metab Dispos.

[22] Velussi M, Cernigoi AM, De Monte A, Dapas F, Caffau C, Zilli M (1997). Long-term (12 months) treatment with an anti-oxidant drug (silymarin) is effective on hyperinsulinemia, exogenous insulin need and malondialdehyde levels in cirrhotic diabetic patients. J Hepatol.

[23] Hajaghamohammadi AA, Ziaee A, Raflei R (2008). The efficacy of silymarin in decreasing transaminase activities in nonalcoholic fatty liver disease. A randomized controlled clinical trial. Hepat Mon.

[24] El-Kamary SS, Shardell MD, Abdel-Hamid M, Ismail S, El-Ateek M, Metwally M (2009). A randomized controlled trial to assess the safety and efficacy of silymarin on symptoms, signs and biomarkers of acute hepatitis. Phytomedicine.

[25] Jacobs BP, Dennehy C, Ramirez G, Sapp J, Lawrence VA (2002). Milk thistle for the treatment of liver disease: a systematic review and meta-analysis. Am J Med.

